# Amitosenescence and Pseudomitosenescence: Putative New Players in the Aging Process

**DOI:** 10.3390/cells8121546

**Published:** 2019-11-29

**Authors:** Diane Wengerodt, Christian Schmeer, Otto W. Witte, Alexandra Kretz

**Affiliations:** 1 Hans-Berger Department of Neurology, Jena University Hospital, 07747 Jena, Thuringia, Germany; diane.wengerodt@med.uni-jena.de (D.W.); christian.schmeer@med.uni-jena.de (C.S.); 2 Jena Center for Healthy Ageing, Jena University Hospital, 07747 Jena, Thuringia, Germany

**Keywords:** cellular senescence, cell cycle, DDR, G_Alert_, postmitotic neurons, replicative reprogramming

## Abstract

Replicative senescence has initially been defined as a stress reaction of replication-competent cultured cells in vitro, resulting in an ultimate cell cycle arrest at preserved growth and viability. Classically, it has been linked to critical telomere curtailment following repetitive cell divisions, and later described as a response to oncogenes and other stressors. Currently, there are compelling new directions indicating that a comparable state of cellular senescence might be adopted also by postmitotic cell entities, including terminally differentiated neurons. However, the cellular upstream inducers and molecular downstream cues mediating a senescence-like state in neurons (amitosenescence) are ill-defined. Here, we address the phenomenon of abortive atypical cell cycle activity in light of amitosenescence, and discuss why such replicative reprogramming might provide a yet unconsidered source to explain senescence in maturated neurons. We also hypothesize the existence of a G_0_ subphase as a priming factor for cell cycle re-entry, in analogy to discoveries in quiescent muscle stem cells. In conclusion, we propose a revision of our current view on the process and definition of senescence by encompassing a primarily replication-incompetent state (amitosenescence), which might be expanded by events of atypical cell cycle activity (pseudomitosenescence).

## The Concept of Neuronal Amitosenescence

The phenomenon of replicative senescence is conventionally defined as a permanent cell cycle arrest in principally replication-competent diploid cells in vitro. This concept, introduced by Hayflick and Moorhead almost 60 years ago [1], still serves as a fundamental guideline to explain senescence also in vivo. Although later expanded to oncogene-induced senescence (OIS) and stress-induced premature senescence (SIPS), the dogma has persisted that neurons are spared from senescence as they do not apply to the basal concepts of the paradigm. Such a view has now been challenged by the discovery that terminally differentiated neurons acquire a senescence-like phenotype as assessed by different markers, such as SA-β-Gal, 4-HNE, γH2AX, mH2A, p-p38, IL-6, and MCP-1 [2,3]. The appearance and features of cellular senescence in postmitotic or rarely dividing tissues, a phenotype recently termed ‘postmitotic cellular senescence’ (PoMiCS) [4] and that we here specify as ‘amitosenescence’ particularly for postmitotic neurons, has been demonstrated in several cell populations, including neuronal cells in tissues of the central nervous system (CNS) [2], cardiomyocytes, osteocytes, and adipocytes [5,6,7]. Current key knowledge regarding this topic has been excellently summarized recently [4].

Causally, the phenomenon of a senescence-like cellular and molecular signature, also implicating morphological changes such as cellular hypertrophy [4] and heterochromatin alterations [2], has been linked to dysfunctional telomeres and a persistent DNA damage response (DDR). These intracellular genomic stressors, however, are now hypothesized to require additional signals, such as for cell expansion, to induce postmitotic senescence [8], which appears entailed by the activation of a senescence-associated secretory phenotype (SASP), as demonstrated by augmented levels of IL-6 and p38 MAPK [2]. Whether a corresponding SASP in neurons might elicit a canonical or rather alternative factor profile, is still ill-defined. In favor of a highly cell type-specific SASP response, both senescent-like cortical neurons and cardiomyocytes apparently differ in their profile of SASP-associated components secreted, as Edn3, Tgfb2, and Gdf15 have been highlighted for cardiomyocytes [5], whereas GATA4 and MCP-1 were identified to support the SASP in cortical neurons [3]. Interestingly, in both senescent-like postmitotic cell entities classical SASP components, such as IL-1α and IL-1β, were not significantly involved [3,5].

Moreover, we recently found that mature non-replicative G_0_ neurons are subjected to an age-related telomere erosion process [9], thus supporting the concept of amitosenescence and a role of telomere attrition in postmitotic senescence. This complements the identification of intratelomeric damage sites as a source of sustained DDR, which do not lead to an overt telomere curtailment [10]. Apart from these first insights into the molecular mechanisms of amitosenescence, including a functional role of p21^Cip1^, which apparently serves as a signal mediator essential to connect DDR to a senescence-like phenotype in neurons [2], other upstream events and molecular downstream cues that might relay senescence-like features to mature neurons are yet to be defined.

## Atypical Neuronal Cell Cycle Activity and Pseudomitosenescence 

**Figure 1:**
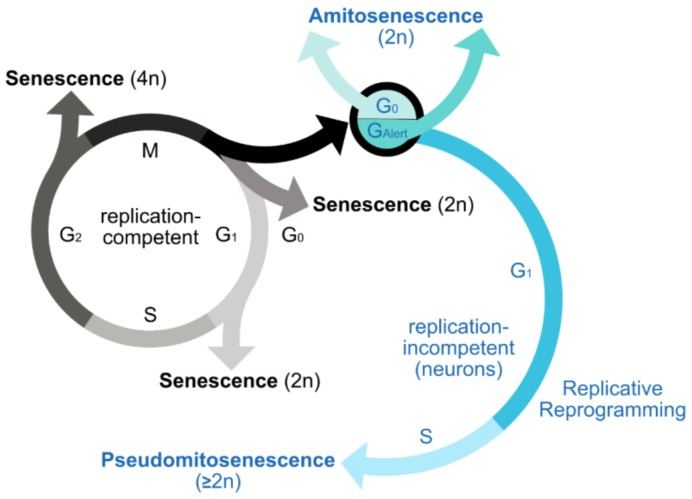
Senescence-like phenotype in neurons and its putative association with cell cycle dynamics. In replicative tissues, senescence is commonly expected to eventuate in G_0_/G_1_; however, it also occurs in the G_2_ phase. According to recent developments in neuroscience, terminally differentiated neurons might also adopt a senescence-like state out of a quiescent G_0_ phase, e.g., consecutive to cellular stress imposed by DNA and telomere damage, a process termed here as ‘amitosenescence’. Whether neurons can further perform an adaptive G_0_–G_Alert_ transition, in analogy to the recent discovery of such an ‘alert state’ in quiescent stem cells, in order to boost respective stress responses, is an interesting, yet unaddressed question. Beyond such novel insight into the conditions of senescence, the long-standing dogma of neurons being ultimately postmitotic has equally been changed. Thus, the reinduction of unscheduled cell cycle activity in terminally differentiated neurons appears broadly established, as illustrated for several neurodegenerative disorders. Though abortive in most instances, such replicative reprogramming might provoke a senescence-inductive DDR due to DNA content variations resulting from a reinitiated S phase. Such a process, termed pseudomitosenescence, might confer additional, yet unidentified cellular and molecular signatures to the process of senescence in neurons. Whether a putative G_Alert_ adaption, which primes quiescent stem cells for cell cycle re-entry, might also prime neurons to complete the G_0_ exit and trigger the reinduction of unscheduled cell cycle activity, is a similarly tempting issue. Grey colors refer to replication-competent cells; blue colors are related to replication-incompetent cells, particularly neurons.

One of these putative inductive mechanisms, which again entails DNA alterations in terms of DNA content variations and thus might also mediate DDR, involves atypical cell cycle reinduction in maturated neurons. Pioneered by the work of Herrup and other groups [11,12], it is now well-established that several stress and neurodegenerative disease conditions accompany the induction of an abortive unscheduled cell cycle in nominally postmitotic neurons. Estimates suggest that up to 13%–40% of inflicted neurons can adopt a state of G_0_ exit [13], paralleled by the upregulation of several Cdks and the suppression of Cdk inhibitors (Cdkls), as indicated by our own studies—a state we therefore term ‘replicative reprogramming’. Such evidence, as well as the recent attempt to force a completed neuromitosis in differentiated cultured neurons [14], suggests that neurons retain a functional cell cycle machinery also in their differentiated state, as illustrated in Figure 1. This endowment includes the activating (Cdks) as well as inhibitory (CdkIs) branch of cell cycle regulators, which can connect atypical cell cycle to both cell cycle progression and apoptosis, and to senescence.

Underlining this interconnection between cell cycle activity and senescence, diseased neurons are well-established to express cell cycle activators, such as Cdk4, coincidentally with p21^Cip1^ and p16^Ink4a^—factors employed to demonstrate a senescent-like condition in postreplicative cell moieties [2,5,15]. In addition, upregulation of cyclinD1, which regulates the progression from G_1_ to S phase, has been specifically assigned to neurons dying by apoptosis [16], an end point of atypical cell cycle reactivation. Moreover, pRb pathways, essentially mediating cell cycle progression, are critically involved in the induction of OIS. In general, phosphorylation of the tumor suppressor Rb through cyclinD:Cdk4/6 activates the E2F transcription factor, thereby propagating cell cycle progression beyond G_1_. In further support of a putative interdependency of replicative reprogramming and senescence in neurons, as indicated in Figure 1, the stress response protein p16^Ink4a^ shows strong binding affinity to the key G_1_ phase regulators Cdk4 and Cdk6 that are specifically inhibited. The main function of Cdk4 and Cdk6 is to permit the transition from G_1_ into S phase of the cell cycle. Thus, in stressed neurons, p16^Ink4a^ might not be restricted to indicate senescence, but mark a compensatory step to prevent atypical cell cycle events in these cells. Moreover, as mentioned above, p21^Cip1^ is regarded as an essential signal transducer between DDR and a senescence-like phenotype in neurons [2]. A p16^Ink4a^ induction in response to the stimulation of Cdk4/Cdk6 and cyclinD1 [15,17] as well as of p21^Cip1^ [2] might, therefore, link atypical cell cycle reactivation to cellular senescence. The direct proof that p16^Ink4a^ and p21^Cip1^ are increased in neurons with signs of replicative reprogramming is currently under investigation. Beside the debated role both of p16^Ink4a^ and p21^Cip1^ in the induction of SASP in replicative cells [18,19], the molecular cues releasing a SASP equivalent in senescent-like neurons still have to be defined.

Notably, it has recently been argued that the expression of p21^Cip1^ in neurons might indicate senescence completely unrelated to cell cycle functions [2]. However, to rule out a putative association with unscheduled cell cycle activity deserves further investigation. Likewise, our hypothesis that cells with features of an atypical cell cycle re-itineration might undergo senescence or be in a presenescent state, a process we here term ‘pseudomitosenescence’, as delineated in Figure 1, is supported by the recent work of Mao and colleagues who directly assigned the upregulation of cyclinD1 to cells with DNA content alterations and a state of G_2_-arrested senescence [17]. Further, direct evidence arises from a study by Casafont and colleagues who illustrated the coincidence of a strong 18-fold upregulation of p21^Cip1^ together with a G_0_–G_1_ transition indicated by a 3.6-fold increase in cylinD1 expression in sensory ganglion neurons exposed to genotoxic stress [20]. Further data are required in order to explore whether cyclinD1 and other cell cycle regulators can serve as a marker for senescence, and in particular of pseudomitosenescence, in terminally differentiated neurons.

One obvious, yet unaddressed, player upstream of p21^Cip1^ is represented by the tumor suppressor p53, which is well-characterized for its role in cell cycle control, DDR, senescence, and apoptosis. Likewise, p53 modulates the mTOR pathway, a master regulator of aging and senescence [21]. Moreover, it exerts suppressive action on the SASP, at least partly by counteracting the activity of p38 MAPK [22]. In the aging rat brain, p53 is upregulated compared to young animals [23]. Moreover, in hiPSCs-derived neurons carrying a loss-of-function mutation of the MECP2 gene causing intellectual disability (Rett syndrome), the mutation-associated induction of p53 is associated with a senescence-like phenotype directly in neurons [24]. With further support, p53 is required for the manifestation of hyperinsulinemia-triggered senescence in terminally differentiated adipocytes [7]. Such lines of evidence render p53 an interesting target, apart from p21^Cip1^, to be evaluated in its function as a mediator of amitosenescence in neurons.

Currently, the notion of p53-dependent amitosenescence in differentiated neurons is unproven. Definition of even more specific molecular and cellular including cell cycle phase conditions might be required to demonstrate a role in the discussed process of pseudomitosenescence. Likewise, in replication-competent fibroblasts, a G_2_ state that fails to further progress through mitosis due to a transient upregulation of p53 is sufficient for induction of p21^Cip1^-APC/C^Cdh1^-pRb-dependent senescence [25,26]. Such observations greatly resemble G_2_ phase neurons subsequent to the initiation of an unscheduled cell cycle, as these neurons will usually also not enter mitosis. Thus, such a concept might possess potential to elucidate the molecular basis of neuronal pseudomitosenescence in future studies.

Beyond classical p53, the p53 protein family comprises the p63 and p73 members, including their diverse isoforms. Aside from an established role of p73 in the survival and maintenance of postnatal and mature neurons [27,28], p73 serves as a cell cycle regulator and genomic stability factor that prevents aberrant DNA replication, e.g., under cyclin–Cdk deregulation in the absence of p53 [29]. Knock out studies have further suggested that p73 is essentially involved in the self-renewal, proliferation, and differentiation of stem and progenitor cells during embryonic and adult neurogenesis in the CNS and in the prevention of their premature senescence [30]. The latter was illustrated by an augmented amount of SA-β-Gal-positive cultured neural stem cells (NSCs) and a strong decrease of mTERT mRNA levels in NSCs derived from neonatal p73^−/−^ mice [30]. More intriguingly, haploinsufficiency for p73 is a hitherto unconsidered susceptibility factor that propagates structural and functional neurodegeneration and histopathologies under CNS aging conditions [31]. In support of our delineated concept, reduced copies of the p73 locus augment the frequency of neurons with a reinduced cell cycle by approximately 200% and propagate the manifestation of a neuronal senescence-like phenotype during aging [31]. However, to definitely prove a role of p73 in pseudomitosenescence requires the demonstration of the coincidence of both, markers of cell cycle reinduction and senescence in these neurons.

Further support for the existence of pseudomitosenescence, the link between atypical cell cycle re-entry and senescence in neurons, arises from the observation that aspects of senescence-associated heterochromatin alterations, the foci formation (SAHF) of which are a key genetic marker of classical senescence, are also found in senescence-like neurons [2]. SAHF, normally absent from quiescent cells, represent specialized segments of heterochromatin that play a particular role in the sequestration of proliferation-promoting and cell cycle regulatory genes such as E2F targets in cells undergoing replicative senescence [32]. Thus, the recently detected concentration of the SAHF marker mH2A in nuclear foci of aged neurons further supports the notion of a preserved ‘dormant’ cell cycle machinery in neurons that requires active and permanent suppression. Thereby, the induction of mH2A might be directly indicative of an altered suppression state under senescence, and reflect a compensatory response to unscheduled cell cycle re-entry in neurons. Therefore, mH2A appears to be a promising marker to prove the concept of pseudomitosenescence in neurons, i.e., the reinduction of an atypical cell cycle as a driver for senescence in differentiated neurons.

In terms of biology, the discovery of a senescent-like phenotype in aged neurons leads to the question about the quantity and importance of these cells for the CNS aging process. In the work by Jurk et al., it was demonstrated that up to 80–90% of neurons in the cortex of highly aged mice become reactive for the senescence marker SA-β-Gal, whereby other markers such as γH2AX and 4-HNE were approximately doubled in their frequency [2]. At young ages, however, 20% of neurons already display a positive signal for γH2AX, and even 40% are reactive for SA-β-Gal [2], a phenomenon that is yet unexplained, but also demonstrated by others [33]. Similar as for other senescence markers, the metabolically derived SA-β-Gal does not specifically indicate senescence, as it can also trace lysosomal activity in neurons. Accounting for such data, it is tempting to speculate that neurons, due to their physiologically postmitotic nature, might be prone to express senescence markers, with yet unresolved biological importance. Thus, whether such markers are reliably indicative of a senescent state, or rather of a physiological postmitotic, but non-senescent condition, is unresolved. As speculated recently, a juvenile/young state of senescence might putatively serve a protective role to prevent stressed neurons and those with DNA injury from apoptosis. However, research on the physiological or pathophysiological role of amitosenescence is only in its infancy and further investigations are required to fully understand this phenomenon.

Apart from the enrichment of metabolically derived aging markers such as SA-β-Gal and lipofuscin, aged neurons are well-known to accumulate DNA damage, a hallmark of senescence, as shown by the age-dependent rise in the frequency of γH2AX in neurons [2]. Cumulative DNA damage and activation of a DDR might thereby arise from the intrinsic limitation of neurons to perform homologous recombination (HR) as the principal strategy to compensate for double strand breaks, reinforced by their high oxygen consumption and ROS production. Though incompetent to perform HR, there is recent evidence that neurons can use an RNA-templated, HR-related but replication-independent DNA recombination repair pathway as alternative DNA reconstituting strategy [34,35]. Thus, in terms of a functional reinterpretation of atypical cell cycle events, it appears tempting to address the question of whether cell cycle reitineration might be repurposed in order to compensate for the limited repertoire of neurons to repair their DNA. Moreover, as DNA damage is a strong inducer of cellular senescence, revisiting atypical cell cycle events in the context of DDR-induced senescence would putatively open up novel insights into how neurons circumvent or execute senescence. Furthermore, it would be equally interesting to evaluate whether elimination of neurons displaying an aberrant cell cycle re-entry, in analogy to current senolytic approaches, will influence the aging process.

In addition to cell cycle regulators, DNA damage and chromatin modifiers, and metabolically active modulators, such as mTORC1, play an established role in the process of senescence. Inhibition of the mTORC1 complex is one of the most effective interventions to counteract aging-related processes and prolong lifespan across different species. It is also involved in multiple morphological and metabolic changes characterizing senescent cells, and it is a key mediator of the SASP response [36]. As it drives geroconversion, it might directly link cellular senescence to the expansion signal postulated for classical- [37] and proposed for postmitotic cellular senescence [8]. Moreover, in quiescent adult stem cells, mTORC1 controls the reversible dynamic shift between a quiescent G_0_ and a recently discovered primed G_Alert_ state, which operates as a sensor state enabling very quick adaption of stem cells to stress and injury, and which primes them for immediate cell cycle entry [38]. According to such lessons from adult stem cells and accumulating evidence that neurons might be indeed dynamic in their cell cycle phases, as illustrated in Figure 1, a corresponding subdivision of the G_0_ phase might also be applicable to neurons. Such step might prepare them to rapidly exit G_0_ and execute cell cycle-related activities such as a repurposed cell cycle re-entry in order to assist DNA damage repair [20], or to accelerate apoptosis at instance of irreparable severe DNA demise. As G_Alert_ is associated with greatly enhanced muscular regeneration after injury [38], a corresponding G_0_ substate in neurons might also support beneficial effects. Similar to stem cells, which actively suppress terminal differentiation, mature neurons also have to continuously and actively repress their cell cycle in order to preserve the reversibility of cell cycle exit. Thus, a G_Alert_ state, which also implicates altered transcriptional activity [38], might principally assure reversibility between different cellular cell cycle states.

Therefore, a yet unaddressed interesting question regarding the establishment of a senescent-like state is in which cell cycle phase neurons might adopt such features. It is generally assumed that terminal growth arrest and senescence of replication-competent cells occurs in G_1_ or G_0_ of the cell cycle. However, this view has been revisited by the recent work of Mao and colleagues who illustrated by FACS-based DNA content analysis that a large moiety of senescent cells, comprising up to 36–60%, arrest in the G_2_ phase [17]. Thus, DNA replication might per se represent a risk factor to drive cells into senescence. Therefore, unscheduled cell cycle propagation, at least up to S phase, might hazard neurons to acquire a senescent-like phenotype. Considering that neurons can adopt a senescent-like state and reactivate atypical cell cycle events to a considerable amount, it would be worth addressing in which cell cycle phase the senescent transformation occurs, and whether a G_Alert_-like phase would predispose cells to re-enter unscheduled cell cycle activity. Identification of biomarkers for a putative G_Alert_-like state in neurons might be a relevant finding as they could indicate a presenescent state, which, by targeted elimination, could lead to the prevention instead of reversion of senescence and aging in postmitotic cell entities.

## Conclusions

In summary, we hypothesize that a reinduced cell cycle in postmitotic cells, which per se can undergo senescence (amitosenescence), might link cellular stress to the induction of a senescent-like but vital state of pseudomitosenescence, and enable neurons to repurpose their preserved cell cycle machinery for alternative HR-independent DNA repair strategies. Further studies are required to prove such notion, and to see whether a putative G_Alert_ state coupled to an atypical cell cycle can be beneficial by retaining DNA damage alert signals and supporting genomic stability.
